# Genome-wide analysis of protein–protein interactions and involvement of viral proteins in SARS-CoV-2 replication

**DOI:** 10.1186/s13578-021-00644-y

**Published:** 2021-07-22

**Authors:** Yiling Jiang, Kuijie Tong, Roubin Yao, Yuanze Zhou, Hanwen Lin, Liubing Du, Yunyun Jin, Liu Cao, Jingquan Tan, Xing-Ding Zhang, Deyin Guo, Ji-An Pan, Xiaoxue Peng

**Affiliations:** 1 The Center for Infection and Immunity Study and Molecular Cancer Research Center, School of Medicine, Sun Yat-Sen University, Guangming Science City, Shenzhen, 518107 China; 2 Nanjing CRYCISION Biotechnology Co., Ltd, Nanjing, 211100 China

**Keywords:** SARS-CoV-2, Interaction map, N, Nsp3, Replication

## Abstract

**Background:**

Analysis of viral protein–protein interactions is an essential step to uncover the viral protein functions and the molecular mechanism for the assembly of a viral protein complex. We employed a mammalian two-hybrid system to screen all the viral proteins of SARS-CoV-2 for the protein–protein interactions.

**Results:**

Our study detected 48 interactions, 14 of which were firstly reported here. Unlike Nsp1 of SARS-CoV, Nsp1 of SARS-CoV-2 has the most interacting partners among all the viral proteins and likely functions as a hub for the viral proteins. Five self-interactions were confirmed, and five interactions, Nsp1/Nsp3.1, Nsp3.1/N, Nsp3.2/Nsp12, Nsp10/Nsp14, and Nsp10/Nsp16, were determined to be positive bidirectionally. Using the replicon reporter system of SARS-CoV-2, we screened all viral Nsps for their impacts on the viral replication and revealed Nsp3.1, the N-terminus of Nsp3, significantly inhibited the replicon reporter gene expression. We found Nsp3 interacted with N through its acidic region at N-terminus, while N interacted with Nsp3 through its NTD, which is rich in the basic amino acids. Furthermore, using purified truncated N and Nsp3 proteins, we determined the direct interactions between Nsp3 and N protein.

**Conclusions:**

Our findings provided a basis for understanding the functions of coronavirus proteins and supported the potential of interactions as the target for antiviral drug development.

**Supplementary Information:**

The online version contains supplementary material available at 10.1186/s13578-021-00644-y.

## Introduction

The severe acute respiratory syndrome coronavirus 2 (SARS-CoV-2), the pathogen for the pandemic of the coronavirus disease 2019 (COVID-19), caused more than 100 million infections and 3 million deaths thus far (WHO). As of now, no anti-SARS-CoV-2 specific drug was available in clinical therapy, except for the FDA’s approval of Veklury (remdesivir), clofazimine and topotecan. Veklury could only shorten the duration of hospitalization of COVID-19 patients if early administration within 48 h of hospital admission was applied [1]. Combined with remdesivir, clofazimine exhibited antiviral synergy in cell culture and animal models [2]. Topotecan suppresses infection-induced inflammation, thus reducing morbidity and rescuing mortality in a transgenic animal model [3]. However, the efficacies of these drugs on patients await further clinical investigation. Vaccines developed using different strategies were authorized by many countries to contain the infection of SARS-CoV-2. However, there are growing concerns about safety like antibody-dependent enhancement (ADE), the duration of the effective anti-SARS-CoV-2 immune response, and the effectiveness against the mutant viruses [4–6]. Thus, the anti-SARS-CoV-2 specific drugs are urgently needed, and more drug targets on this deadly virus are waiting to be uncovered.

SARS-CoV-2, the newest member of the genus *Betacoronavirus* of the *Coronaviridae* family, encapsulated the known largest single-stranded positive-sense viral RNA, which encoded a 7000-aa polyprotein for its replicase complex and a set of accessory proteins [7–10]. The polyprotein is encoded by the open reading frame (ORF) 1a and 1ab, and the latter is translated via the mechanism of -1 ribosomal frameshifting. The ORF1a and ORF1ab are processed by the viral proteases, papain-like protease (PLpro) and 3C-like Protease (3CLpro), into 16 non-structural proteins (Nsps), which compose the replicase complex through an unknown manner. The other 10 ORFs encodes 4 well-known structural proteins, spike (S), nucleocapsid (N), membrane (M) and envelope (E), and a set of accessory proteins without clearly identified functions, ORF3, ORF6, ORF7a, ORF7b, ORF8, and ORF10 [9].

As the largest RNA virus, coronaviruses employ a relatively big number of viral proteins to replicate and transcript their viral RNA and subgenomic RNAs compared with other RNA viruses. This strategy increased the efficiency in many aspects of viral proliferation, including replication fidelity of viral RNAs and viral accessory proteins’ expression. However, the performance of this strategy is highly dependent on the coordination of at least 16 viral non-structural proteins, indicating the disturbance on the assembly of viral protein complexes could be a promising approach to the inhibition of viral replication [11].

The direct interactions among viral proteins play essential roles in the formation of viral replication and transcription complex (RTC), assembly of viral particles, release from the host cells and counter-defense against host immune responses. Compared with the active centers, which are mostly inside the structure of proteins, these interactions happen at the peripheral residues of viral proteins. They are likely more vulnerable to small molecules which could competitively bind to the residues which mediate viral protein–protein interactions [12, 13].

Several studies focusing on the interactions between viral proteins and the interactions between viral proteins and host proteins were carried out and uncovered many interactions [14–16]. Immunoprecipitation in combination with mass spectrometry (IP-MS) and yeast two-hybrid (Y2H) screening were employed in these studies. IP-MS is unable to differentiate indirect interactions from direct ones. It usually needs a relatively high amount of target proteins and thus is likely not sensitive enough to detect weak or transient interactions. The interactions identified by Y2H could be determined to be direct, while due to the different intracellular environment from mammalian cells, some interactions, which happen specifically in mammalian cells, could be missed using Y2H screening [17].

Our previous work employed a mammalian two-hybrid system to screen genuine protein interactions for SARS-CoV and identified a few interactions that were not described in other studies using IP-MS or Y2H [17]. Many novel interactions, like the interactions of Nsp10/Nsp14 and Nsp10/Nsp16, uncovered in our previous studies, were proven to play essential roles in the replication of viral genomic RNAs.

In this study, we used a mammalian two-hybrid system to examine 784 interaction combinations between 28 SARS-CoV-2 proteins in a pairwise matrix. As a result, 48 interactions were detected, and 14 interactions of 48 have not been reported elsewhere. We identified 19 interactions between Nsps and accessory proteins. To determine the possible roles of accessory proteins involved in viral replication, we investigated the interaction between Nsp3.1 and N proteins. We found the N interacted with the N-terminus of Nsp3.1 through its N-terminus domain, and disruption of the interaction harms viral replication and transcription. We also explored the roles of all the viral Nsps in viral replication and transcription and identified Nsp3.2 proteins play positive roles in viral replication and transcription.

## Results

### Identification of protein–protein interactions of SARS-CoV-2 using mammalian two-hybrid system assays

**Fig. 1:**
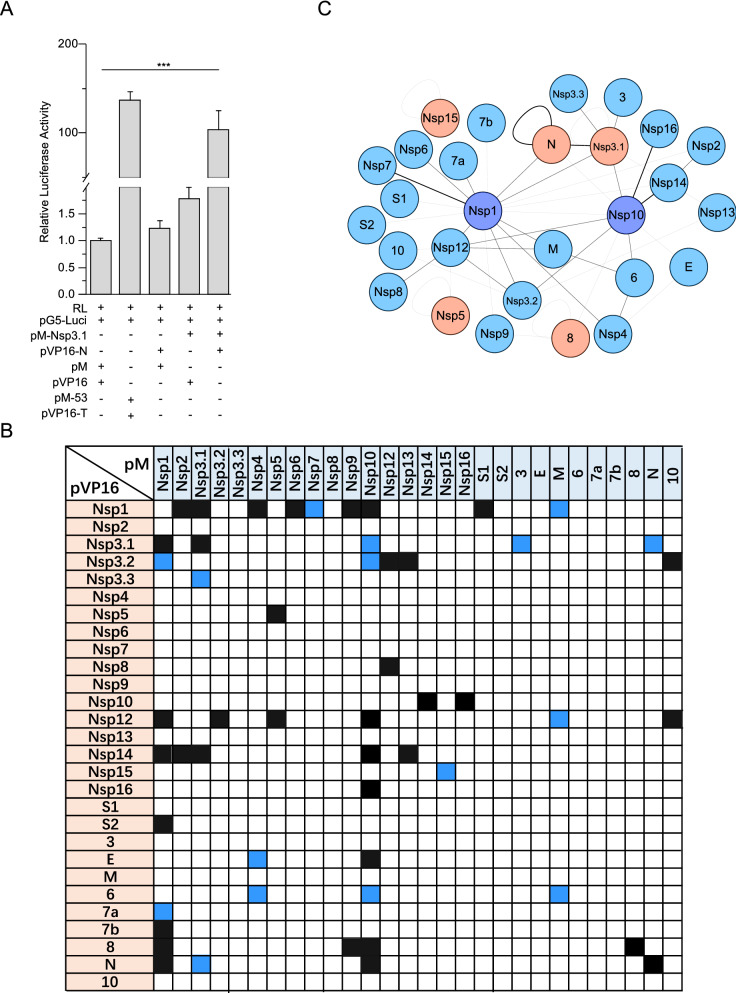
Protein interactions of SARS-CoV-2 detected using mammalian two-hybrid assays. **A** A representative result of positive interaction. The combination of pM-53 and pVP16-T was used as the positive control. **B** Interaction matrix of SARS-CoV-2 proteins. Black squares indicate the interactions reported previously, and blue squares indicate the novel interactions detected in this study. **C** The interactions were analyzed with Cytoscape. The darker blue circles indicated that Nsp1 and Nsp10 had more interacting partners than the blue circles-labeled proteins. The red circles indicate that the proteins had self-interactions. The sticks linked different circles depicted the interactions, and the thickness of the sticks was correlated with the strength of interactions, which was judged arbitrarily based on the results of mammalian two-hybrid assays. Data represent one of 3 independent experiments with similar results; error bar represent mean ± s.e.m; *P < 0.05, **P < 0.01, ***P < 0.001; two-tailed unpaired Student’s t-test

To analyse the genome-wide protein–protein interactions of SARS-CoV-2, We cloned all the coding sequences of Nsps and ORFs into the pM and pVP16 vectors separately (Table 1). To achieve a better expression, we separated the coding sequence of Nsp3 into 3 parts, Nsp3.1, Nsp3.2, and Nsp3.3, which was designed according to the known functional domains of Nsp3 (Additional file 1: Figure S1). S was separated at the cleavage site of furin into S1 and S2. The expression of all the viral proteins in both pM and pVP16 vectors were examined, and the expressions of 11 proteins in pM vectors and 9 proteins in pVP16 were confirmed using immuno-blotting (Additional file 1: Figure S2). Despite that the expressions of some proteins were not detected, the interactions, such as Nsp16/Nsp10, were still detected, indicating that the mammalian two-hybrid system is sensitive enough to detect interactions between proteins, which were not expressed well. The sensitivity and efficiency of the mammalian two-hybrid system were also confirmed by detecting the interaction of p53 and SV40T, which could lead to more than 130 times of increase in the relative expression level of reporter genes compared with negative controls (Fig. 1A).

**Table 1:** The sequences of SARS-CoV-2 used for interaction analysis

Protein	Coding sequence*	Position in polyprotein*	Protein length	Function described**
Nsp1	266–805	M1-G180	180	Inhibits host gene expression
Nsp2	806–2719	A181-G818	638	
Nsp3.1	2720–4966	A819-T1568	749	ADRP, SUD for OGB
Nsp3.2	4967–7105	I1569-T2282	713	PLpro, DU
Nsp3.3	7106–8554	Y2283-G2765	483	
Nsp4	8555–10054	K2766-Q3266	500	Membrane rearrangement, essential for viral replication
Nsp5	10055–10972	S3267-Q3572	306	3C-like proteinase
Nsp6	10973–11842	S3573-Q3863	290	TM
Nsp7	11843–12091	S3864-Q3945	81	dsRNA-binding
Nsp8	12092–12685	A3946-Q4144	198	dsRNA-binding & RdRP
Nsp9	12686–13024	N4145-Q4258	113	ssRNA-binding
Nsp10	13025–13441	A4259-Q4398	139	GFL
Nsp12	13442–16236	S4399-Q5331	932	RdRP
Nsp13	16237–18039	A5332-Q5933	601	The helicase enzyme, NTPase
Nsp14	18040–19620	A5934-Q6461	527	ExoN
Nsp15	19621–20658	S6462-Q6808	346	EndoRNase, degrades viral dsRNA
Nsp16	20659–21552	S6809-N7107	298	2ʹ-*O*-MT
S1	21563–23617	n/a	685	Spike (receptor-binding)
S2	23618–25384	n/a	588	Spike (fusion peptide & TM)
3	25393–26220	n/a	275	TM, ion channel, antagonist of IFN
E	26245–26472	n/a	75	envelope
M	26523–27191	n/a	222	Membrane
6	27202–27387	n/a	61	Viral pathogenesis
7a	27394–27759	n/a	121	
7b	27756–27884	n/a	43	
8	27894–28259	n/a	121	Disrupts antigen presentation
N	28274–29533	n/a	419	Binds viral RNA, antagonize antiviral RNAi
10	29558–29674	n/a	38	

*n/a* not applied

*The coordinate of the sequence is based on the genome of severe acute respiratory syndrome coronavirus 2 isolate Wuhan-Hu-1 (NCBI Reference Sequence: NC_045512.2)

**Abbreviations: *ADRP* adenosine diphosphate-ribose 1”-phosphatase, *SUD* SARS Unique Domain, *OGB* oligo(G)-binding, *PLpro* papain-like cysteine proteinase, *DU* deubiquitinating activity, *TM* transmembrane domain, *RdRP* RNA-dependent RNA polymerase, *GFL* growth-factor-like protein, *NTPase* NTP and RNA 5’ triphosphatase, *ExoN* 3’ to 5’ exonuclease, *2’-O-MT* S-adenosylmethionine-dependent ribose 2’-O-methyltransferase, *IFN* interferon

**Fig. 2:**
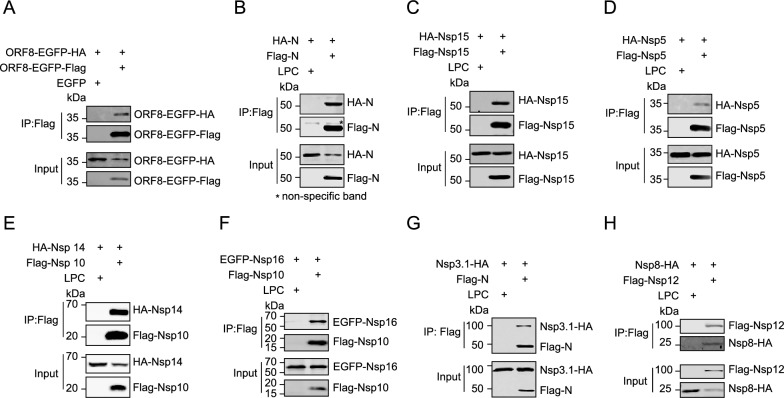
Confirmation of protein interactions by co-immunoprecipitation. The coding sequences of each viral proteins fused with Flag or HA tag were cloned into LPC vector, respectively. The combination of two proteins with indicated tags was expressed in HEK293T cells, and the cell lysates were collected for co-immunoprecipitation with Flag agarose. The samples from co-immunoprecipitation were examined with WB, and the two proteins were detected with Flag and HA antibodies. ORF8 was fused with EGFP for an increased expression level. Four self-interactions, ORF8 (**A**), N (**B**), Nsp15 (**C**), and Nsp5 (**D**), and Four interactions between various viral proteins, Nsp14-Nsp10 (**E**), Nsp16-Nsp10 (**F**), Nsp3.1-N (**G**), and Nsp8-Nsp12 (**H**) were confirmed using co-immunoprecipitation. Data represent one of 3 independent experiments with similar results

We screened 784 interaction combinations between all the coding sequences of Nsps or ORFs of SARS-CoV-2, and the assays on each combination were repeated at least three times (Fig. 1B). 48 positive interaction combinations were screened out and analyzed using Cytoscape software to visualize molecular interaction networks (Fig. 1B, C). Some interactions were confirmed using co-immunoprecipitation (Fig. 2). Besides 5 self-interactions, 5 out of 48 interactions between different viral proteins, Nsp1/Nsp3.1, Nsp3.1/N, Nsp3.2/Nsp12, Nsp10/Nsp14, and Nsp10/Nsp16, were examined to be positive bidirectionally, and 79.2% interactions could only be detected in one direction, indicating that the fusion domains may influence the interactions, which happened in our previous studies [17]. We identified 14 novel interactions, including Nsp1/Nsp7, Nsp1/M, Nsp1/Nsp3.2, Nsp1/ORF7a, Nsp3.1/Nsp10, Nsp3.1/ORF3, Nsp3.2/Nsp10, Nsp3.3/Nsp3.1, Nsp12/M, E/Nsp4, ORF6/Nsp4, ORF6/Nsp10, ORF6/M, and Nsp3.1/N.

Unlike the Nsp1 of SARS-CoV, which barely interacts with other viral proteins, Nsp1 of SARS-CoV-2 has the most interaction partners, 9 Nsps and 7 accessory proteins, among all the viral proteins, and likely functions as the hub of the group of viral proteins (Fig. 1B, C). As the first viral protein being expressed after viral infection, the function of Nsp1 as a hub likely was an advantage for virus to organize the assembly of replicase complex and replication of viral genomic RNAs. Besides its function in suppression of protein expression and defense against host innate immune response [18], the vital role of Nsp1 in the viral replication is supported by the clinical observation that a deletion in the C-terminus of Nsp1 attenuated the viral pathogenicity [19]. Nsp3 is one of the most complex proteins and has multiple functional domains [20]. The well-known function of Nsp3 is to process viral polyprotein at cleavage sites of Nsp1/2, Nsp2/3 and Nsp3/4. In our interaction screening, Nsp3 was found to interact with Nsp10, Nsp12, Nsp13, and Nsp14, indicating its possible roles in the replication/transcription of viral RNAs besides the process of viral polyprotein. Nsp5 is responsible for the cleavage at the sites separating Nsp4 to Nsp16, spanning 1a and 1b regions. Similar to SARS-CoV, Nsp5 of SARS-CoV-2 interacts with Nsp12, indicating that Nsp12 could be the sites in 1b for Nsp5 to grasp its substrate [17]. In agreement with the previous findings that Nsp8 and Nsp12 form the core RNA polymerase complex, Nsp8 of SARS-CoV-2 interacts with Nsp12 [21, 22]. As an RNA binding protein, Nsp8 may facilitate the substrate recognition of Nsp12. We identified 9 interactions Nsp10 involved in, and among them, the interactions of Nsp10/Nsp14 and Nsp10/Nsp16 were also uncovered in our previous work for SARS-CoV [17], indicating a conservative mechanism of the genus *Betacoronavirus* for regulation of the activity of methyltransferase of Nsp14 and Nsp16. We also identified many interactions between Nsps and accessory proteins, indicating that the possible roles of accessory proteins in the replication/transcription of viral RNAs. 5 self-interactions, Nsp3, Nsp5, Nsp15, and N reported here were also found in SARS-CoV, except for ORF8, which is one of the most distinct ORFs between SARS-CoV and SARS-CoV-2 [23, 24]. We failed in screening out the several known interactions, such as Nsp7/Nsp12 and Nsp7/Nsp8. The replication and transcription complex (RTC) of SARS-CoV-2 is assembled in a double membrane structure in the ER-Golgi complex. This special viral membrane environment could be different from the nuclear compartments, where the two-hybrid system works. The folding of the protein could be impacted by these different environments, thus leading to various behaviors in the interaction with another proteins. Therefore, the capacity of the mammalian two-hybrid system could be impacted by the micro-environment of the different intracellular loci.

### Nsp3 interacts with N protein

N protein is one of four well known structural proteins identified in the viral particles [7]. It forms a long helical nucleocapsid structure in which viral RNA was packed inside. This ribonucleoprotein (RNP) complex protects the viral RNA from the attack of host nucleases and recognition of host nucleotide sensors triggering the immune response [25]. This structure could also play an essential role in the replication/transcription of viral RNA [17]. However, the molecular details about the role of N in replication were obscure. In this screening, the interaction between Nsp3.1 and N was among the strongest ones (Fig. 1A) and was confirmed by co-immunoprecipitation (Fig. 2G). Since Nsp3 is the component of the viral replication and transcription complex (RTC), this interaction suggested the N could regulate the replication of viral RNA through the association with Nsp3.

### N protein interacts with Nsp3 through its NTD domain

**Fig. 3:**
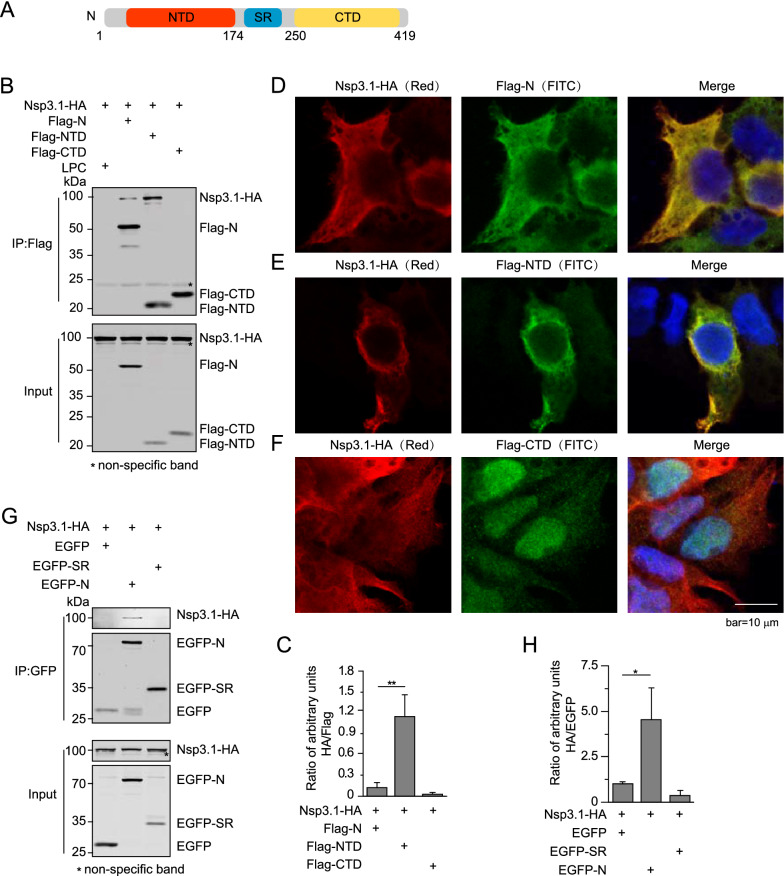
N interacts with Nsp3 through its NTD. **A** Schematic diagram of SARS-CoV-2 N protein structure. HEK293T cells were transfected with the indicated combinations of plasmids. The cells subjected to co-immunoprecipitation with Flag agarose (**B**) and immunostaining with Flag and HA antibodies (**D**–**F**). **C** The intensities of HA or Flag stained bands of each sample were quantified using LI-COR Image Studio software, and ratios of the intensities of HA/Flag bands were calculated. Note that compared with N, NTD interacted with Nsp3.1 in a stronger manner. Similarly, the interaction between Nsp3.1 and SR of N was examined using co-immunoprecipitation with GFP antibody (**G)**. Their interactions were quantified by calculating the ratios of intensities of HA/EGFP bands (**H**). Note that SR of N lost the capacity to interact with Nsp3.1. Data represent one of 3 independent experiments with similar results; error bar represent mean ± s.e.m; *P < 0.05, **P < 0.01, ***P < 0.001; two-tailed unpaired Student’s t-test

N protein has three major defined domains, N-terminal domain (NTD), serine-arginine-rich (SR) domain, and C-terminal domain (CTD) (Fig. 3A) [26, 27]. To determine N protein’s key domains interacting with Nsp3.1, we examined the interactions between various domains of N and Nsp3.1 using co-immunoprecipitation. NTD retains the capability to interact with Nsp3.1, while this capability of N protein is largely lost in CTD (Fig. 3B, C). We also examined the locations of N and Nsp3.1 proteins in the cells. The immunostaining results showed that similar to the wild-type (wt) N, NTD colocalized with Nsp3.1 in the 293T cells, while CTD lost the colocalization with Nsp3.1 (Fig. 3D–F). We could not detect the expression of the coding sequence of SR, which happens typically in the expression of proteins with a molecular weight of less than 15 kDa. In our laboratory practice, we increase the size of protein by fusing our target protein with a tag protein, like EGFP, which has a relatively independent structure and unlikely interferes with the function of target protein. As predicted, we detected the decent expression of EGFP-tagged SR. However, Nsp3.1-HA could not be detected in the pull-down samples of EGFP-tagged SR, similar as that of EGFP, while, in contrast, the decent level of Nsp3.1-HA was detected in that of EGFP-tagged N (Fig. 3G, H). Moreover, NTD seems to interact with Nsp3.1 in a stronger manner than N protein, indicating that the other domains of N protein could negatively impact the interaction with Nsp3.1 (Fig. 3C).

### Nsp3 interacts with N through its acidic domain

**Fig. 4:**
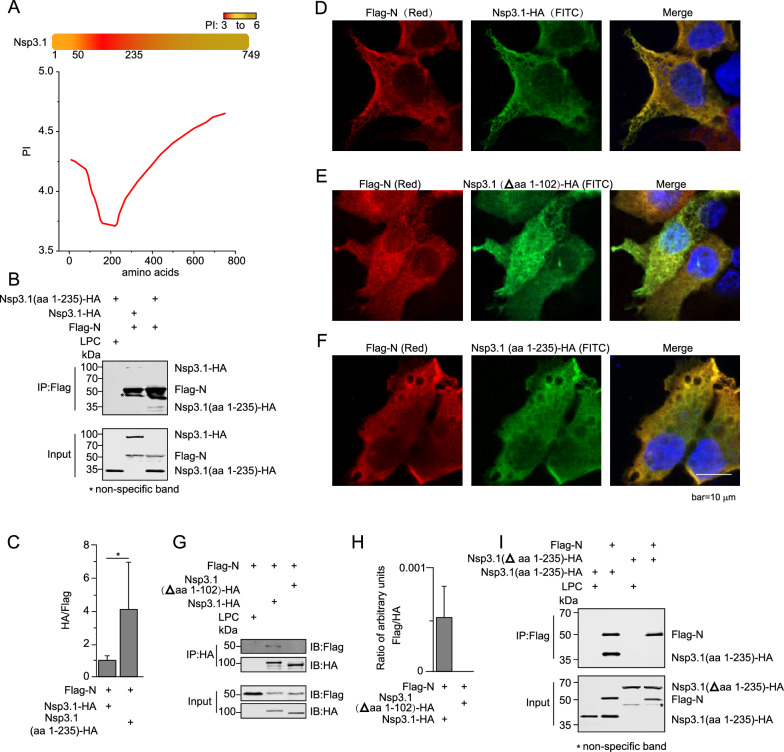
Nsp3.1 interacts with N through its N-terminal domain. **A** Schematic diagram of PI values of various Nsp3.1 regions generated using Expasy ProtParam tool. Note that a strong acidic region (aa 1–235) is located in the N-terminus of Nsp3.1. HEK293T cells were transfected with the indicated plasmid combinations. The cells were subjected to co-immunoprecipitation with Flag agarose (**B**) and immunostaining with Flag and HA antibodies (**D**) and (**E**). **C** The intensities of HA or Flag stained bands of each sample were quantified using LI-COR Image Studio software, and ratios of the intensities of HA/Flag bands were calculated. Note that compared with Nsp3.1 (aa 1–749 of Nsp3), aa 1–235 interacted with N in a stronger manner. Similarly, the interaction between N and Δaa 1–102 (**G**) or Δaa 1–235 (**I**) of Nsp3.1 was examined using co-immunoprecipitation with HA antibody and immunostaining with Flag and HA antibodies (**F**). Their interactions were quantified by calculating the ratios of intensities of Flag/HA bands (**H**). Note that deletion of aa 1–102 impaired the capacity of Nsp3 to interact with N. Data represent one of 3 independent experiments with similar results; error bar represent mean ± s.e.m; *P < 0.05, **P < 0.01, ***P < 0.001; two-tailed unpaired Student’s t-test

Thus far, limited knowledge about the functions and structures of Nsp3.1 was available. Based on analysis of the protein sequence, we found a special domain rich in negatively charged amino acids in the N-terminus of Nsp3.1 (Fig. 4A). As a nucleic acid-binding protein, N protein has a 10.1 of pI and is composed of many positively charged amino acids, which facilitate its interaction with nucleic acids with negative charges and likely also with acidic proteins (Additional file 1: Figure S3). Accordingly, we first examined the interaction between N protein and the aa 1–235 of the N-terminus, which possessed the most acidic amino acids in Nsp3.1. Despite the loss of nearly two-thirds of Nsp3.1, aa 1–235 retained the capability to interact with N protein (Fig. 4B, C). To further narrow down the region that interacts with N protein, we removed aa 1–102, which has the most acidic amino acids, from Nsp3.1 and examined its interaction with N protein. As predicted, the deletion of aa 1–102 largely abolished Nsp3.1/N interaction (Fig. 4G, H). By observing the colocalizations between N and truncated Nsp3.1, we confirmed that only aa 1–235 of Nsp3.1 plays an indispensable role in the interaction between Nsp3.1 and N (Fig. 4D–F). To further confirm the dependency of aa 1–235, we deleted aa 1–235 in Nsp3.1 and found the deletion abolished the interaction between Nsp3.1 and N (Fig. 4I).

### Nsp3 and N formed a protein complex in vitro

**Fig. 5:**
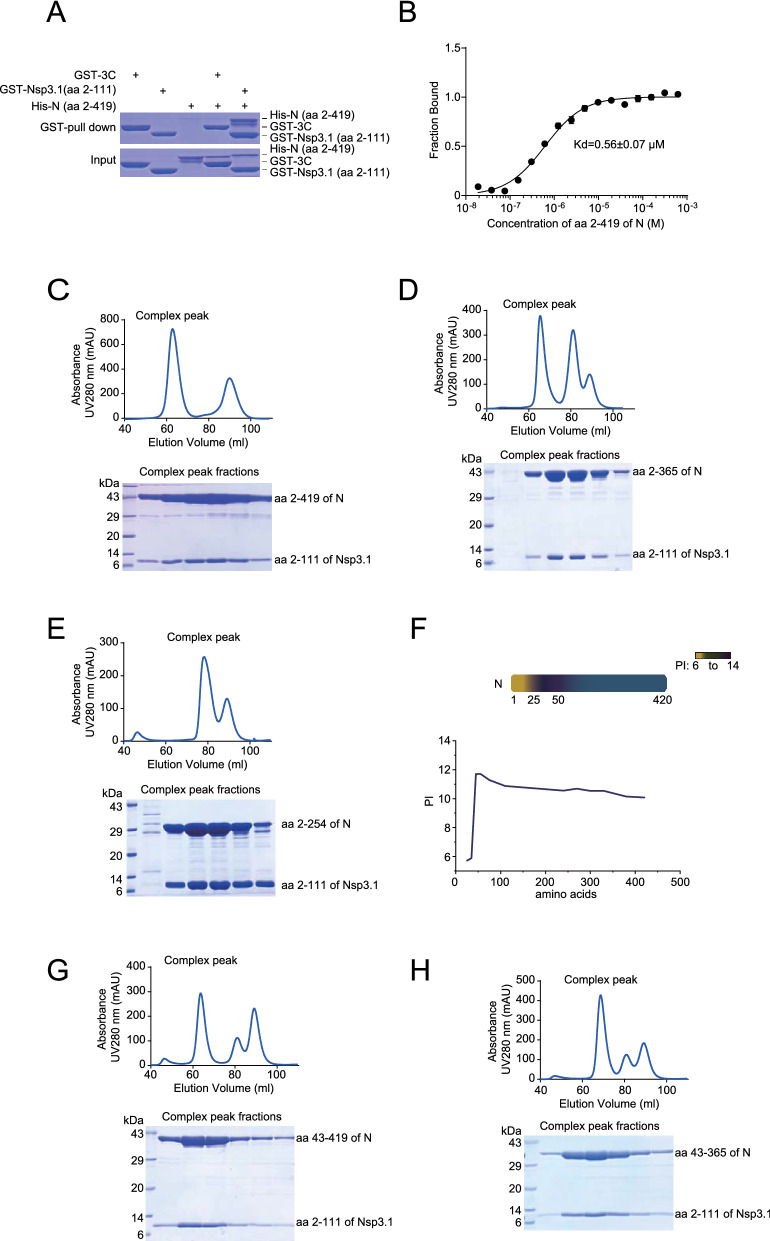
Confirmation of interactions between truncated N and Nsp3 proteins using purified proteins. The aa 2–111 (**A**) and aa 2–180 of Nsp3.1 (**B**) and truncated N proteins aa 2–419 (**A**), aa 2–365 (**D**), aa 2–254 (**E**), aa 43–419 (**G**) and aa 43–365 (**H**) were fused with glutathione *S*-transferase (GST) and 6xHis tag at the N-terminus, respectively. The aa 2–111 and aa 2–180 of Nsp3.1 and various truncated N proteins were co-expressed in *Escherichia coli* BL21 (DE3). The cell lysates were mixed with glutathione Sepharose-4B beads, washed and eluted with lysis buffer containing 15 mM reduced glutathione. The elutes were digested with PreScission protease, and undigested proteins were cleaned with glutathione Sepharose-4B beads. **A** The interaction between GST-Nsp3.1 (aa 2–111) and His-N (aa 2–419) was verified using GST-pull down assay. **B** The interaction affinity of aa 2–180 of Nsp3.1 and aa 2–419 of N protein was quantified using MST. The proteins without tags were analysed by gel filtration, and the fractions around the peak related to the protein complex were examined using SDS-PAGE and coomassie blue staining (**C**–**E**, **G** and **H**). **F** Schematic diagram of PI values of various N regions generated using Expasy ProtParam tool

Next, we sought to confirm N could directly interact with Nsp3. Since aa 1–102 of Nsp3.1 was indispensable for N-Nsp3.1 interaction, we used aa 2–111 of Nsp3.1 instead of Nsp3.1 to check the interaction in vitro. We expressed and purified GST-Nsp3.1 (aa 2–111) and His-N (aa 2–419) from bacteria and performed GST-pull down assay, verifying their interactions (Fig. 5A). To quantify the interaction affinity, we performed a microscale thermophoresis (MST) assay, which showed aa 2–111 of Nsp3.1 bound to aa 2–419 of N protein with a dissociation constant (Kd) of 0.56 ± 0.07 μM (Fig. 5B). To further investigate the potential of complex formation of these two proteins, we co-expressed aa 2–419 of N and aa 2–111 of Nsp3.1 proteins in the bacteria, and the two proteins were purified together. Gel filtration analysis showed aa 2–419 of N and aa 2–111 of Nsp3.1 migrated together. Through the conversion of elution volume to the approximate molecular weight, at least two aa 2–419 of N proteins could be in the fraction of complex peak, indicating that the interaction sites between Nsp3 and N should be different from the sites for the formation of the oligomer of N and that of Nsp3 (Fig. 5C), and the recognition of replicase complex on N through Nsp3 should not disturb the structure of N oligomers. Consistent with our IP results, partial (Fig. 5D) or complete (Fig. 5E) deletion of CTD retained the interaction with Nsp3.1.

Sequence analysis indicated that 43 basic amino acids at the N-terminus of N were likely dispensable for the interaction if the N-Nsp3.1 interaction depended on the attraction between the acidic and basic domains (Fig. 5F). Indeed, gel filtration analysis confirmed that the deletion of 43 aa did not impact the interactions (Fig. 5G, H).

### The interaction between Nsp3 and N protein played an essential role in the replication and transcription of viral genomic RNAs

As the direct interacting protein with viral genomic RNAs, N protein composed the nucleocapsid structure wrapping viral RNA and thus could be involved in viral replication and transcription [7, 17]. Nsp3 is processed from the viral polyprotein, which composed RTC and thus it could also join in the viral replication and transcription. Although both could play important roles in viral replication and transcription, whether the association of RTC and N of SARS-CoV-2 was essential for viral replication and transcription was not defined.

**Fig. 6:**
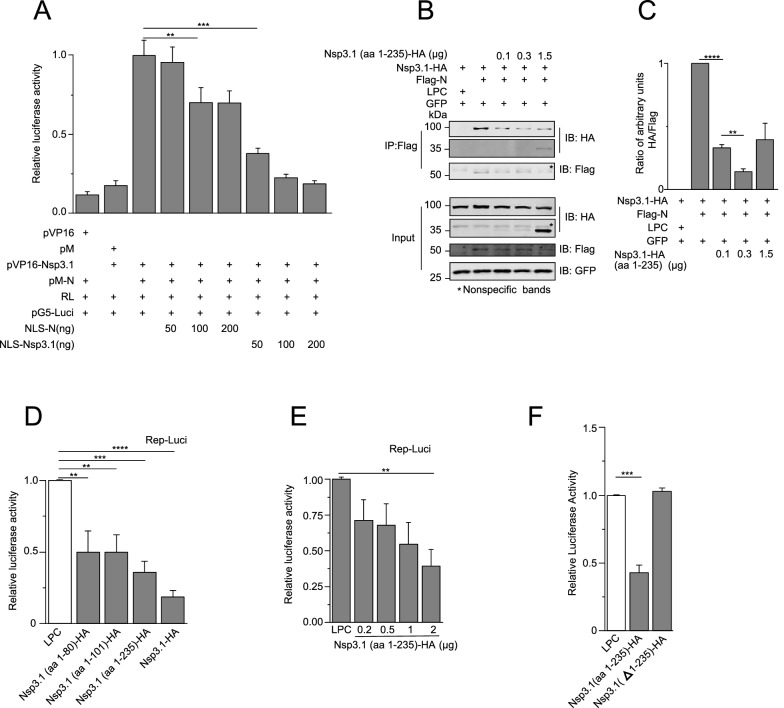
Inhibition of Nsp3-N interaction in *trans* impaired viral replication. **A** HEK293T cells were transfected with pVP16-Nsp3.1 expressing AD-Nsp3.1, pM-N expressing BD-N, nuclear-localized Nsp3.1 (NLS-Nsp3.1), and nuclear-localized N (NLS-N). 36 h post-transfection, the cells were subjected to the Dual-Glo Luciferase Assay. Note that in a dose-dependent manner, NLS-N or NLS-Nsp3.1 inhibited the expression of luciferase, the reporter gene in pG5-Luc, promoted by the interaction of AD-Nsp3.1 and BD-N. **B** HEK293T cells were transfected with Nsp3.1-HA, aa 1–235 of Nsp3.1-HA, Flag-N, LPC vector, and EGFP as an indicator for transfection effection. 36 h post-transfection, the cells were subjected to the co-immunoprecipitation assay and WB analysis. **C** The intensities of HA or Flag stained bands of each sample were quantified using LI-COR Image Studio software, and ratios of the intensities of HA/Flag bands were calculated. Note that in a dose-dependent manner, aa 1–235 of Nsp3.1 competed with Nsp3.1 for the interaction with N. HEK293T cells were transfected with Rep-Luci, RL, and various truncated Nsp3.1 proteins (**D**–**F**). 48 h post-transfection, the cells were subjected to the Dual-Glo Luciferase Assay. Note that all truncated Nsp3.1 proteins except Δ1-235 of Nsp3.1 (**F**) inhibited the replication of replicon of SARS-CoV-2, and the aa 1–235 of Nsp3.1 inhibited the replication of replicon in a dose-dependent manner (**E**). Data represent one of 3 independent experiments with similar results; error bar represent mean ± s.e.m; *P < 0.05, **P < 0.01, ***P < 0.001, ****P < 0.0001; two-tailed unpaired Student’s t-test or one-way ANOVA.

We investigated whether inhibition of the interaction between Nsp3 and N could influence the replication and transcription of viral genome. Firstly, we determined whether the interaction could be inhibited. To this end, we constructed the N or Nsp3.1 fused with Nuclear Localization Sequence (NLS). Next, we investigated whether NLS-N or NLS-Nsp3.1 could disturb the interaction between BD-N and AD-Nsp3.1 in the nucleus. Indeed, both NLS-N or NLS-Nsp3.1 could inhibit the interaction between BD-N and AD-Nsp3.1 in a dose-dependent manner (Fig. 6A). We also confirmed the aa 1–235 of Nsp3.1 could compete with Nsp3.1 in the interaction with N protein using co-immunoprecipitation (Fig. 6B, C).

Since the limited availability of biosafety level 3 (BSL3) laboratory, we utilized the viral replicon instead of live SARS-CoV-2 as a model to study the impact of inhibition of the interaction between Nsp3.1 and N on the viral replication (Additional file 1: Figure S4A). The replicon of SARS-CoV-2 (nCoV-replicon) constructed by our lab expressed the S gene-deleted full-length RNA of viral genome. The deletion of S gene abolished the generation of viruses which raised concerns of biosafety. We replaced the coding sequence of S gene with the reporter gene firefly luciferase, whose expression is driven by the transcription regulatory sequence (TRS) of S gene, and the replicon with firefly luciferase is named as Rep-Luci (Additional file 1: Figure S4A). We verified the activity of the replicon by quantifying the expression of subgenomic RNAs of viral genes (Additional file 1: Figure S4C). The mutations of S759A/D760A/D761A (SDD) in nsp12, which impaired the RdRP activity, abolished the activity of replicon, indicating that the activity of firefly luciferase could reflect the level of replication and transcription of the replicon (Additional file 1: Figure S4C).

To investigate the role of viral Nsps on viral replication and transcription, we expressed plasmids expressing viral Nsps, Rep-Luci and RL-TK plasmids in 293 T cells and measured the relative luciferase activities (Additional file 1: Figure S4B). Many viral Nsps promoted the activity of Rep-Luci, but Nsp3.1 inhibited the activity and the expression of subgenomic RNAs of replicon, indicating its potential as an inhibitor for viral replication (Additional file 1: Figure S4B and S4D). We further examined the inhibitory effect of aa 1–80, aa 1–101, or aa 1–235 of Nsp3 on the activity of Rep-Luci (Fig. 6D). All of the truncated Nsp3.1 proteins, as well as full-length Nsp3.1 protein, inhibited the replication and transcription of replicon, and the inhibitory effect of aa 1–235 of Nsp3 was in a dose-dependent manner (Fig. 6E). Except that the inhibitory effects of aa 1–80 and aa 1–101 of Nsp3.1 protein were comparable, the inhibitory effects increased in the order of aa 1–101, aa 1–235, and the full-length of Nsp3.1. Interestingly, the coverage of the region of Nsp3.1 of these truncated mutants was also increased in this order. To further confirm the dependency of aa 1–235, we deleted aa 1–235 in Nsp3.1 and found the deletion abolished the inhibitory effect of Nsp3.1 on the replicon activity (Fig. 6F). The co-immunoprecipitation results showed that the interaction between aa 1–235 and N was much stronger than that between wt Nsp3.1 and N, indicating that Nsp3.1 could inhibit the function of Nsp3 independent of its interaction with N. As the truncated form of natural Nsp3 protein, Nsp3.1 could exhibit a dominant-negative effect through inactivation of the other function of wt Nsp3 protein.

## Discussion

As one of the largest RNA viruses, SARS-CoV-2 encodes at least 26 proteins or peptides. Except some of them may function independently, many of them should form protein complex to regulate the replication/transcription of viral genome RNA, the assembly of viral particles, escape from the recognition of host immune defense system, and other functions[7, 18]. Protein–protein interactions play important roles in the processes mentioned above, and thus the disruption of the critical interactions could result in the inhibition of viral proliferation. The drugs developed on these targets are virus-specific, and unlikely inhibit the functions of host cells. COVID-19 has spread worldwide for more than a year, and anti-SARS-CoV-2 specific drugs were urgently needed. The new essential interactions for viral replication will be helpful for viral drug developments.

An independent intraviral protein–protein interactome of SARS-CoV-2 finished recently by Liang group uncovered 58 interactions using yeast two-hybrid and co-immunoprecipitation. Using the different system, we also identified the 34 interactions which were reported by Liang group. Due to the different strategy for screening the protein–protein interactions, we identified 14 interactions which were not detected in the study of Liang group. Among the 14 interactions, the self-interaction of ORF8 and Nsp3-N interaction were as strong as some known combinations, such as Nsp10-Nsp14/Nsp16. This further indicated that the proteins might behave differently in various cell contexts, and thus the interactions that happened in mammalian cells are likely not to be detected in yeast and other cell systems. Indeed, in our previous work, we reported six novel viral protein–protein interactions of SARS-CoV, which were not uncovered in the other studies using yeast system.

Compared with other RNA viruses, SARS-CoV-2 uses a more complex mechanism to replicate and transcribe its viral genome. 16 non-structural proteins and at least one protein encoded by the ORFs in the 3ʹ-proximal end of the viral genome were known involved in this mechanism. Non-structural proteins were processed from the same polyproteins, and thus, their coordination was likely set up while being expressed. As the main interaction protein of viral genome RNA, N protein was believed to play an important role in viral replication and transcription, despite that molecular details for this role were still obscure.

Therefore, after establishing the intraviral protein–protein interactome, we focused on the interactions between Nsps and N protein. In our interaction map, the interaction between N protein and Nsp3.1 was tested to be positive in both directions. The interactions were also confirmed by co-immunoprecipitation after the expression of two proteins in the mammalian cells and by gel filtration using the purified proteins from bacterial cells.

If Nsp3 was the linker for N to join in the viral RTC, the disruption of this interaction would inhibit the replication/transcription of viral genomic RNAs. To reveal more details, we examined the interactions between different domains of N and Nsp3.1 proteins. We found that N interacts with Nsp3.1 through its N-terminal domain, which contains the most basic amino acids of N protein. The interaction domain in Nsp3.1 is also at its N-terminus, which contains the most acidic amino acid of Nsp3.1 protein. Our results showed the Nsp3.1-N interaction could perform in a manner of charge attraction, indicating a less site-specificity. We found the interaction between NTD and Nsp3.1 was stronger than that of N and Nsp3.1, and CTD, composed of basic residues, negatively impacted the interaction. We hypothesized that CTD itself was incompetent to interact with Nsp3.1 and its basic residues could interfere with the interaction between NTD and Nsp3.1 in a competitive manner.

Due to the limited availability of P3 lab, we used the viral replicon of SARS-CoV-2 to investigate the inhibitory effect of truncated Nsp3.1 on viral replication/transcription. As predicted, the truncated Nsp3.1, which interacted with N protein, markedly decreased the replication/transcription of the replicon. However, Nsp3.1 exhibited a more substantial inhibitory effect than truncated Nsp3.1, indicating that as the truncated Nsp3, Nsp3.1 not only disrupted the interaction between Nsp3 and N but also inhibited some unknown functions of Nsp3. In our design, Nsp3.1 and Nsp3.2 were separated at the linker between Domain Preceding Ubl2 and PL2pro (DPUP) and the ubiquitin-like domain 2 (Ubl2). The inhibitory effect of Nsp3.1 suggested DPUP and Ubl2 mutually regulated their functions in a cis manner, and trans regulator could only exert a dominant-negative regulatory effect on the fusion protein of DPUP and Ubl2. The cis mutual regulation between DPUP and Ubl2 could be the potential target for anti-SARS-CoV-2 design.

To obtain more details about the interaction between Nsp3.1 and N, We co-expressed various truncated Nsp3.1 and N proteins and purified their complex using gel filtration. Although we obtained crystals containing various complexes, the resolutions were not good enough to determine the accurate structures. Since the interaction between Nsp3 and N protein was dependent on electrostatic forces, we hypothesized that the formation of the complex was in a dynamic process, and the complex’s stability was relatively low. Therefore, it is essential to stabilize the complex’s structure using some means before taking the structure’s snapshot.

## Conclusion

We used a mammalian two-hybrid system to identify the intraviral protein–protein interactions. Together with the previous studies performed by other groups, we established the relative complete intraviral protein interactome. To further validate the potential of interactions as the target for antiviral drug development, we selected Nsp3-N interactions, which was not reported previously, for further investigation and confirmed that the potential of Nsp3-N interaction as the target to inhibit the replication of SARS-CoV-2.

## Materials and methods

### Cell culture

HEK293T cells were cultured in Dulbecco’s Modified Eagle’s Medium (DMEM), supplemented with 10% FBS, 100 units/mL penicillin, and 100 µg/mL streptomycin (Thermo Fisher Scientific) at 37 °C with 5% CO_2_.

### Plasmid construction and transfection

The sequences of open reading frames (ORFs) and Nsps were amplified with primers containing/not containing a sequence of Flag or HA tag using Gold mix (TSINGKE) and cloned into the desired vectors. All clones were validated using Sanger sequencing (TSINGKE).

Hieff Trans™ Liposomal Transfection Reagent (Yeasen) was used for transfection. One day before transfection, 1 × 10^5^ cells were plated in 48-well plate. One hour before transfection, the medium was replaced with DMEM without supplements. The plasmids and liposome were incubated in Opti-MEM (Thermo Fisher Scientific) for 5 min before mixed at a ratio of 1:2 (μg:μL). 20 min post-incubation, the mixtures were added in the cell culture dropwise. 6 h post-transfection, the media were replaced with a complete medium. Two days after culture, the cells were subjected to the downstream assays.

### Immunoprecipitation and immunoblot analysis

For immunoprecipitation, cells were collected and lysed in 500 µL DISC IP lysis buffer (50 mM Tris–HCl, pH 7.5, 150 mM NaCl, 10% glycerol and 1% Triton X-100), supplemented with protease inhibitor cocktail (Roche, 1:100 of dilution), 1 mM PMSF, 1 mM NaVO_4_. After centrifugation at 12,000*g* for 10 min at 4 °C, the supernatant was recovered. 80 μL of the sample was saved as ‘Input’ control and stored at − 80 °C. The remaining supernatant was incubated with agarose conjugated with appropriate antibodies and was rotated overnight at 4 °C. The next day, the agarose was washed three to five times in DISC IP washing buffer for 10 min each time at 4 °C. The samples bound to the agarose were eluted in 50 µL of the elution buffer (0.1 M Glycine–HCl, pH 3.5) for 5 min and then neutralized in 10 µL of neutralization buffer (0.5 M Tris–HCl, pH 7.4). All samples were stored at − 80 °C.

The samples’concentration was measured using BCA assay (Thermo Fisher Scientific) and bored with 2 × SDS loading buffer for 5 min at 100 °C. Proteins were separated by SDS-PAGE and then transferred to Nitrocellulose (NC) membranes. Membranes were blocked in PBST (0.1% Tween-20 in PBS) containing 5% skim milk and then incubated overnight with indicated primary antibodies at 4 °C. The next day, membranes were washed five times with PBST and incubated with appropriate secondary antibodies for 45 min at room temperature. Then, membranes were washed four to five times with PBST. The final blots were scanned and quantified using Odyssey® CLx Imaging System (LI-COR Biosciences). Primary antibodies used were anti-HA (1:1000 for WB, Biolegend), anti-Flag (1:1000 for WB, Sigma), and anti-GFP (1:1000 for WB, Proteintech). The secondary antibodies were goat anti-rabbit IRDye 800CW and goat anti-mouse IRDye 680RD (1:10,000 for WB, LI-COR Biosciences).

### Protein expression and purification

The fragment of Nsp3.1 (aa 2–243, aa 3–180, aa 3–111 and aa 106–180) and N protein (aa 2–419, aa 2–175, aa 2–203, aa 2–254, aa 2–365, aa 43–365 and aa 43–419) were cloned into a pGEX-6p-1 vector with GST tag and a pRSFDuet-1 vector with a 6xHis tag and a PreScission protease site (LEVLFQ’GP) at the N-terminus, respectively. The Nsp3.1-N protein complex was co-expressed in *Escherichia coli* BL21 (DE3). Briefly, the overnight cultures were transferred into fresh LB medium containing 50 μg/mL kanamycin and 100 μg/mL ampicillin, and induced with 0.1 mM IPTG when OD600 reached to 0.8 and cultured at 20℃ about 16 h. The cells were harvested by centrifugation, and the pellets were resuspended in lysis buffer (50 mM Tris–HCl, pH 8.0, 300 mM NaCl, 10% glycerol and 5 mM MgCl_2_). The cells were then disrupted by the high pressure cracker (UH-24, Union-biotech), and cell debris was removed by centrifugation. The supernatant was mixed with glutathione Sepharose-4B beads (GE Healthcare), and rocked for 2 h at 4 °C. Subsequently, the glutathione Sepharose-4B beads were transferred into a column and washed with lysis buffer about 10 volumes. Then, the protein was eluted with lysis buffer containing 15 mM reduced glutathione. The protein products were digested with PreScission protease and dialysis against reduced glutathione in lysis buffer to rebind the glutathione Sepharose-4B beads. The Nsp3.1-N protein complex was mainly collected in flow-through sample, and maybe few GST tag contaminant could be detected. Finally, the Nsp3.1-N protein complex was further purified and analysed by gel filtration chromatography. The fractions were collected and subjected to SDS-PAGE, followed by coomassie blue staining.

### Microscale thermophoresis assay

The binding affinity of aa 2–180 of Nsp3.1 and aa 2–419 of N was measured by using the Monolith NT.115 (Nanotemper Technologies). Aa 2–180 of Nsp3.1 was fluorescently labelled according to the manufacturer’s procedure (RED-NHS MO-L011) and kept in the MST buffer (50 mM Tris–HCl, pH 8.0, 250 mM NaCl, 10 mM MgCl2, 0.05% Tween-20) at a concentration of 100 nM. Next, the RED fluorescent dye NT-647 was added, mixed and incubated for 30 min at 25 °C in the dark. For each assay, the labelled aa 2–180 of Nsp3.1 was mixed with the same volume of unlabeled aa 2–419 of N at 16 serially diluted concentrations at room temperature. The samples were then loaded into premium capillaries and measured at 25 °C by using 40% LED power and medium MST power. The assay was repeated three times. Data analyses were performed using MO. Affinity Analysis v.2.2.4 software. There is 95% confidence that the Kd value is within the given range. All data were processed using GraphPad Prism 8.0.2.

### GST-pull down assay

Recombinant glutathione S-transferase (GST)-Nsp3.1 (aa 2–111) and His-N (aa 2–419) were expressed in *Escherichia coli* BL21 (DE3), respectively. Bacterial cells were harvested and lysed by sonication in ice. GST-Nsp3.1 (aa 2–111) was immobilized on the glutathione-Sepharose, and then His-N (aa 2–419), which was purified using Ni–NTA Agarose Beads, were loaded on the resin of glutathione-Sepharose. After incubation with rotation for 2 h, the resin of glutathione-Sepharose was washed by PBS at least for five times. The resin was resuspended in SDS-PAGE loading buffer and heated at 100 °C for 5 min. The samples were separated in 12% SDS-PAGE and stained with Coomassie Brilliant Blue.

### Mammalian two-hybrid and dual-luciferase reporter assays

The details of the assay were described previously [17]. In brief, cells in each well of 48-well plate were transfected with 300 ng DB fusion genes in pM, 300 ng AD fusion genes in pVP16, 100 ng pG5-Luci as reporter construct and 100 ng pRL-TK (Promega) as an internal control.

48 hours post-transfection, the cells were washed with PBS and lysed in 100 µL of passive lysis buffer (PLB). 20 µL of cell lysate from each sample was subjected to Dual-Glo Luciferase Assay System (Promega) according to the manufacturer’s instructions. The values of firefly luciferase and renilla luciferase were measured in Synergy H1 Hybrid Multi-Mode Microplate Reader (Biotek) and the ratios of the two luciferases’ values were calculated to obtain the relative luciferase activity. A total of 784 assays, 28 × 28, were performed and each assay was repeated 3 times at least. The combination of pM and AD fusion genes in pVP16 and that of pVP16 and BD fusion genes in pM were used as negative controls.

### Immunofluorescence

HEK293T cells were seeded on coverslips in 24-well plate. 48 h post-transfection with indicated plasmids, cells in each well were washed with 200 μL PBS and fixed with PBS containing 4% paraformaldehyde at room temperature for 15 min. Then cells were washed once with PBS and permeabilized with PBS containing 0.1% Triton X-100 at room temperature for 10 min. After being washed with PBS and PBST (PBS containing 0.1% Tween), cells were incubated in 500 µL of PBST containing 5% goat serum (blocking buffer) for 1 h and then left in a blocking buffer containing indicated antibodies overnight at 4 °C. The next day, the cells were washed 4 times in PBST at room temperature for 10 min each time and then incubated in a blocking buffer containing appropriate secondary antibodies for 1 h. After being washed three times with PBST and one time with PBS, the cells were stained with DAPI solution (1 μg/mL DAPI in PBS) for 5 min and left in PBS. The coverslip was mounted on slides with ProLong™ Gold Antifade Mountant (Thermo Fisher Scientific). The slides were observed under Nikon Eclipse Ti2E (Tokai Hit STX stagetop incubator).

### RNA extraction and reverse transcription

TRIzol® Reagent (Thermo Fisher Scientific) was used to extract total RNA from cells. Briefly, 0.5 mL of TRIzol reagent was added to cells in 6 cm plate. After 5 min of incubation, 0.1 mL chloroform was added to the lysate. The sample was mixed thoroughly and centrifuged for 15 min at 12,000×*g* at 4 °C. The clear aqueous phase was recovered and mixed with 0.25 mL of isopropanol. After 10 min of incubation at 4 °C, the RNA was precipitated using 10 min of centrifugation at 12,000×*g* at 4 °C. The RNA pellet was washed once with 70% ethanol and dissolved in RNase-free water after air-dry. 1 μg of total RNA was reversely transcribed with oligo(dT) primer using the PrimeScript RT reagent Kit with gDNA Eraser (TaKaRa).

### Statistics

Two-tailed Student's t-test or one-way ANOVA were used to analyse the significance of the differences between two or more groups. Results were considered significant when p-value was less than 0.05.

## Supplementary Information

**Additional file 1: Figure S1.** The conserved domains of Nsp3 of SARS-CoV-2. The diagram of Nsp3 with indicated conserved domains was produced according to the description of YP_009725299.1 of CDD of NCBI. The separation sites aa 749 for Nsp3.1 and aa 1462 for Nsp3.2 were labelled in the diagram. **Figure S2.** The expression of all the pM and pVP16 constructs. HEK293T cells were transfected with the indicated pM (A) and pVP16 (B) constructs. 36 h post-transfection, the cells were collected and subjected to WB, and the blots were stained with indicated antibodies. **Figure S3.** Acid–base analysis for the viral proteins encoded by SARS-CoV-2. Viral proteins encoded by SARS-CoV-2 were listed in the order of PI values. Note that N is the most basic protein and Nsp3.1 is among the most acidic proteins of SARS-CoV-2. **Figure S4.** The regulatory effect of various Nsps on the replicon of SARS-CoV-2. The cDNA of SARS-CoV-2 genome was inserted in the BAC vector. Its expression was driven by the human Cytomegalovirus (CMV) promoter upstream of its sequence. The bovine growth hormone polyadenylation (BGH) was employed to terminate the transcription and was removed by a hepatitis delta virus ribozyme (RZ) to generate the authentic 3’ terminal sequence of SARS-CoV-2. The S gene was replaced with firefly luciferase to eliminate the generation of live virus and to quantify the replication of replicon. (B) HEK293T cells were transfected with the plasmids expressing indicated viral Nsps, Rep-Luci, and pRL-TK. 48 h post-transfection, the cells were collected and subjected to the Dual-Glo Luciferase Assay. Note that Nsp3.1 drastically inhibited the activity of Rep-luci, the replicon of SARS-CoV-2. (C) HEK293T cells were transfected with Rep-Luci or Rep-Luci with mutations of S759A/D760A/D761A (SDD) in nsp12. 48 h post-transfection, the cells were collected and subjected to quantitative RT-PCR with the common forward primer in leader sequence and indicated reverse primers in various genes at the 3’ proximal end of SARS-CoV-2. Note that deficiency of Nsp12 abolished the expression of subgenomic RNAs of the viral replicon. (D) HEK293T cells were transfected with viral replicon and the plasmids expressing Nsp3.1, Nsp12 or N protein. 48 h post-transfection, the cells were collected and subjected to quantitative RT-PCR for indicated subgenomic RNAs. Note that Nsp3.1 drastically inhibited the subgenomic RNA expression of the viral replicon.

## Data Availability

All data generated or analysed during this study are included in this published article and its supplementary information files.
