# Supplementary material for: Genome-wide analysis of protein–protein interactions and involvement of viral proteins in SARS-CoV-2 replication
Source: Cell Biosci. 2021 Jul 22;11:140. doi: 10.1186/s13578-021-00644-y (PMC8295636; doi:10.1186/s13578-021-00644-y)
Supplement: Supplementary file 1 — Additional file 1: Figure S1. The conserved domains of Nsp3 of SARS-CoV-2. The diagram of Nsp3 with indicated conserved domains was produced according to the description of YP_009725299.1 of CDD of NCBI. The separation sites aa 749 for Nsp3.1 and aa 1462 for Nsp3.2 were labelled in the diagram. Figure S2. The expression of all the pM and pVP16 constructs. HEK293T cells were transfected with the indicated pM (A) and pVP16 (B) constructs. 36 h post-transfection, the cells were collected and subjected to WB, and the blots were stained with indicated antibodies. Figure S3. Acid–base analysis for the viral proteins encoded by SARS-CoV-2. Viral proteins encoded by SARS-CoV-2 were listed in the order of PI values. Note that N is the most basic protein and Nsp3.1 is among the most acidic proteins of SARS-CoV-2. Figure S4. The regulatory effect of various Nsps on the replicon of SARS-CoV-2. The cDNA of SARS-CoV-2 genome was inserted in the BAC vector. Its expression was driven by the human Cytomegalovirus (CMV) promoter upstream of its sequence. The bovine growth hormone polyadenylation (BGH) was employed to terminate the transcription and was removed by a hepatitis delta virus ribozyme (RZ) to generate the authentic 3’ terminal sequence of SARS-CoV-2. The S gene was replaced with firefly luciferase to eliminate the generation of live virus and to quantify the replication of replicon. (B) HEK293T cells were transfected with the plasmids expressing indicated viral Nsps, Rep-Luci, and pRL-TK. 48 h post-transfection, the cells were collected and subjected to the Dual-Glo Luciferase Assay. Note that Nsp3.1 drastically inhibited the activity of Rep-luci, the replicon of SARS-CoV-2. (C) HEK293T cells were transfected with Rep-Luci or Rep-Luci with mutations of S759A/D760A/D761A (SDD) in nsp12. 48 h post-transfection, the cells were collected and subjected to quantitative RT-PCR with the common forward primer in leader sequence and indicated reverse primers in various genes at the [file 13578_2021_644_MOESM1_ESM.pdf]

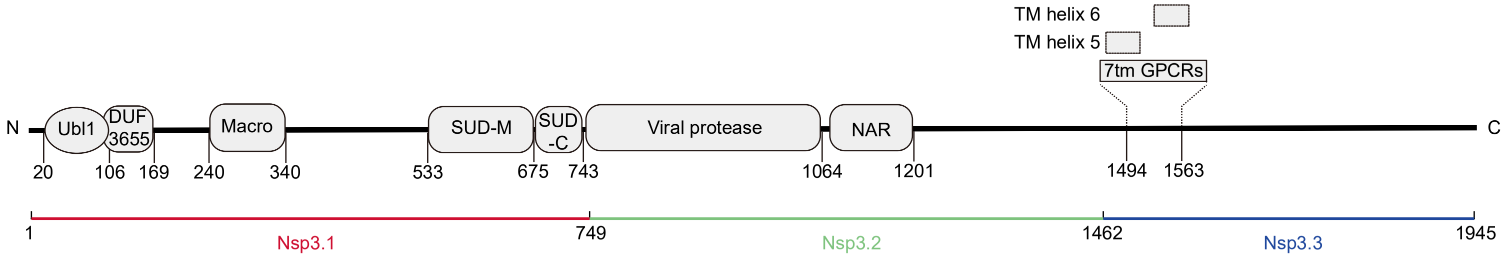

The Nsp3 of SARS-CoV-2 （ref. YP_009725299.1). Ubl1, ubiquitin-like domain 1; DUF3655, protein of unknown function; SUD-M, single-stranded poly(A) binding domain; SUD-C, C-terminal SARS-Unique Domain; Viral protease, papain like viral protease; NAR, nucleic acid-binding domain; 7tm GPCRs, seven-transmembrane G protein-coupled receptor superfamily. TM helix 5 and 6 are structural motifs.

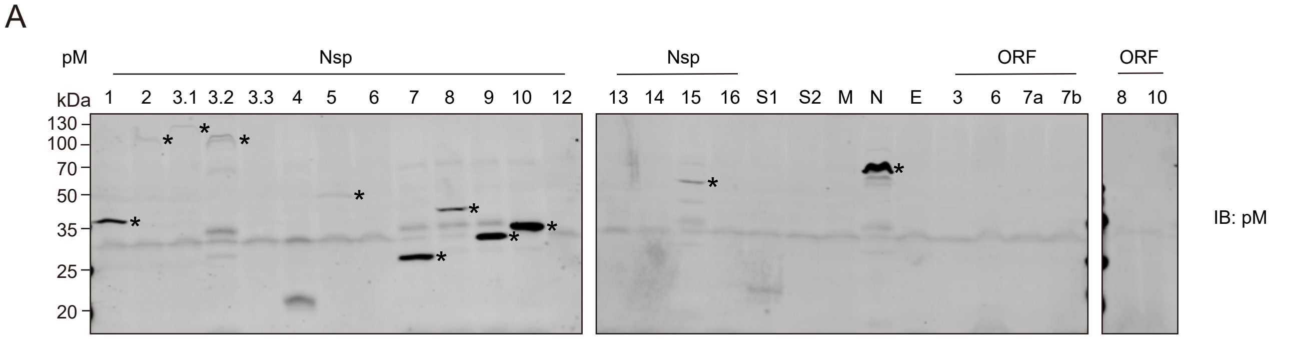

*Viral protein with the predicted size

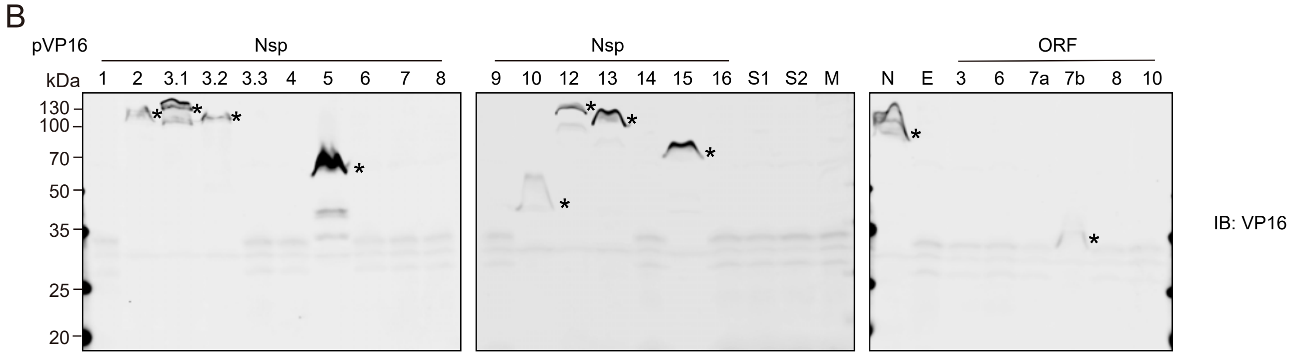

*Viral protein with the predicted size

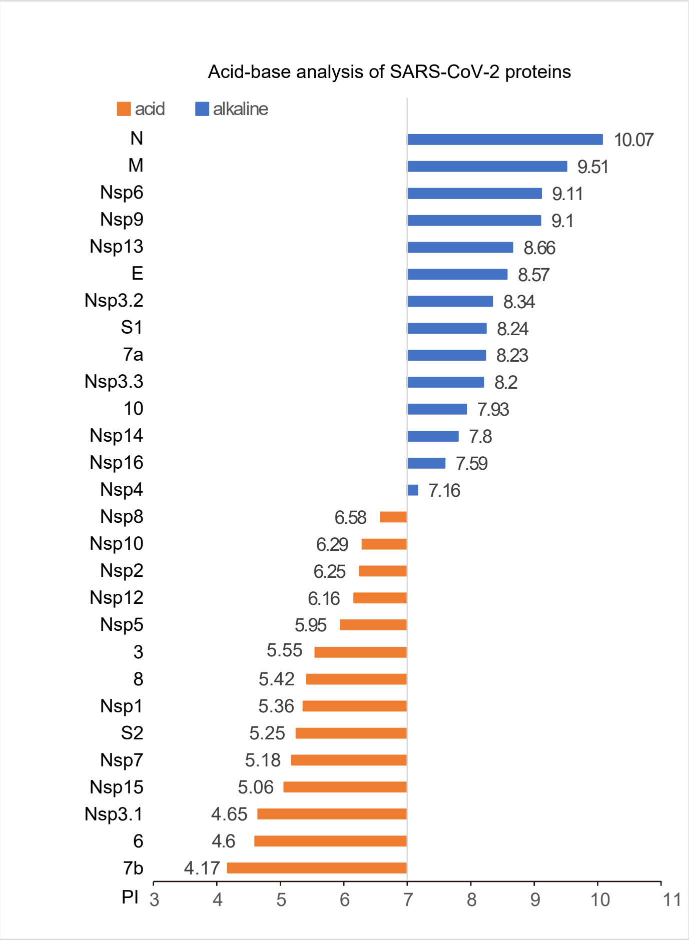
Acid-base analysis of SARS-CoV-2 proteins
acid
alkaline
N
M
Nsp6
Nsp9
Nsp13
E
Nsp3.2
S1
7a
Nsp3.3
10
Nsp14
Nsp16
Nsp4
Nsp8
Nsp10
Nsp2
Nsp12
Nsp5
3
8
Nsp1
S2
Nsp7
Nsp15
Nsp3.1
6
7b
10.07
9.51
9.11
9.1
8.66
8.57
8.34
8.24
8.23
8.2
7.93
7.8
7.59
7.16
6.58
6.29
6.25
6.16
5.95
5.55
5.42
5.36
5.25
5.18
5.06
4.65
4.6
4.17
PI
3
4
5
6
7
8
9
10
11

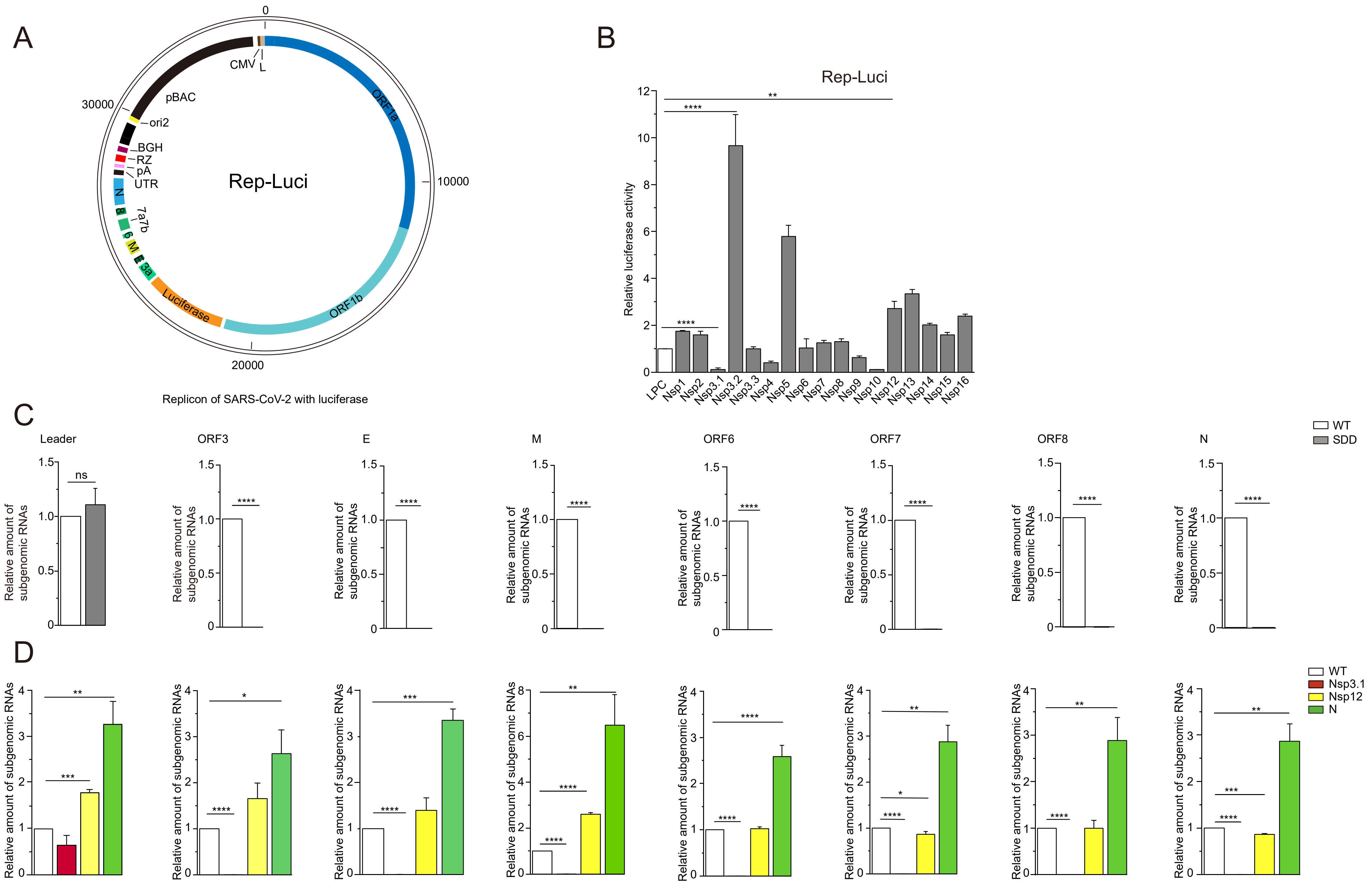
A
0
CMV
L
ORF1a
10000
ORF1b
20000
Luciferase
3a
E
M
6
7a7b
8
N
UTR
pA
RZ
BGH
ori2
pBAC
30000
Rep-Luci
Replicon of SARS-CoV-2 with luciferase
B
Rep-Luci
Relative luciferase activity
0
2
4
6
8
10
12
****
**
LPC
Nsp1
Nsp2
Nsp3.1
Nsp3.2
Nsp3.3
Nsp4
Nsp5
Nsp6
Nsp7
Nsp8
Nsp9
Nsp10
Nsp12
Nsp13
Nsp14
Nsp15
Nsp16
C
Leader
ORF3
E
M
ORF6
ORF7
ORF8
N
Relative amount of subgenomic RNAs
0
0.5
1.0
1.5
ns
****
WT
SDD
D
Relative amount of subgenomic RNAs
0
1
2
3
4
6
8
*
**
***
****
WT
Nsp3.1
Nsp12
N
